# The Interplay Between Parental Bonding and Health-Related Quality of Life in Kidney Transplant Recipients: A Cross-Sectional Study

**DOI:** 10.3390/jcm14134673

**Published:** 2025-07-02

**Authors:** Maria Luisa Pistorio, Concetta De Pasquale, Vittorio Lenzo, Massimiliano Veroux, Magy Martin, Don Martin, Alessia Giaquinta, Martina Giambra, Pierfrancesco Veroux, Maria Catena Ausilia Quattropani

**Affiliations:** 1 Vascular Surgery and Organ Transplant Unit, Department of General Surgery and Medical-Surgical Specialties, University Hospital of Catania, 95123 Catania, Italy; alessia.giaquinta@unict.it (A.G.); martinagiambra@gmail.com (M.G.); pveroux@unict.it (P.V.); 2 Department of Educational Sciences, University of Catania, 95123 Catania, Italy; vittorio.lenzo@unict.it (V.L.); maria.quattropani@unict.it (M.C.A.Q.); 3 Organ Transplant Unit, Department of Surgical and Medical Sciences and Advanced Technologies, University Hospital of Catania, 95123 Catania, Italy; mveroux@libero.it; 4 School of Psychology, Walden University, Minneapolis, MN 55401, USA; magy.martin@mail.waldenu.edu; 5 Private Practice, Youngstown, OH 15009, USA; drdonmartin1@gmail.com

**Keywords:** clinical psychology, kidney transplantation, parental bonding, quality of life, attachment, mental health

## Abstract

**Background:** Few studies have investigated the impact of parental bonding on the quality of life and psychological health in kidney transplant recipients. Exploring these factors could provide valuable insights into the development of psychosocial interventions aimed at improving patients’ psychological adjustment and their overall quality of life. In this perspective, our study aimed to explore how dimensions of parental bonding, particularly maternal care and overprotection, may influence the quality of life and psychological well-being in kidney transplant recipients. By investigating these relationships, the study seeks to understand whether early maternal attachment experiences can predict psychological outcomes in adult transplant recipients. **Methods:** A cross-sectional study involving a sample of 99 kidney transplant recipients (69.7% males, mean age  =  52  ±  9.93 years) was conducted. Participants were recruited from the outpatient clinic of an Italian transplant center between May 2022 and July 2024. After an initial telephone interview, 1-2 interviews were performed in person to administer the questionnaires of the established protocol: the Parental Bonding Instrument (PBI) to identify the type of parental bond and the Short Form-36 (SF-36) Health Survey to evaluate the quality of life perceived by the patients. **Results:** Regression analyses revealed that higher perceived maternal care during childhood was positively associated with better psychological health during adulthood (*β* = 0.290; *p* < 0.05). Conversely, higher levels of perceived maternal overprotection were negatively associated with psychological health in this population (*β* = −0.286; *p* < 0.05). **Conclusions:** The results suggest that maternal affection and support may serve as a protective factor, while excessive maternal protection could impair the development of emotional coping mechanisms necessary for dealing with the stresses of adult life.

## 1. Background

Quality of life (QoL), defined as a complex set of physical, psychological, and social factors, represents a particularly relevant parameter for patients who have undergone a kidney transplant [1].

Kidney transplantation is the optimal therapy for chronic kidney disease, allowing most patients to regain a good quality of life. It is important to achieve an optimal balance between graft function and the patient’s QoL [2].

While kidney transplantation offers a significant improvement in renal function and can prolong life, it is not without psychological challenges. Transplant recipients face numerous emotional stressors related to chronic illness, the side effects of immunosuppressive drugs, the risk of organ rejection, and concerns about the longevity of the transplant [3]. In the study by Golenia [4], a prevalence of 12.5% for depression and 27% for anxiety was found in kidney transplant recipients who had been transplanted for at least 35 months; furthermore, patients who took higher doses of prednisone suffered more from these symptoms. Shdaifat [5] found a worse mental component of quality of life among hemodialysis patients and kidney transplant recipients compared to peritoneal dialysis patients.

While advances in immunosuppressive therapy have improved graft and patient survival, it is uncertain whether this objective success is also reflected in the subjective well-being of patients. Kidney transplant recipients experience varying degrees of disease-specific physical and psychological impairment, some of which are attributed to the adverse effects of immunosuppressants. Affective disorders, somatoform disorders, and emotional distress may arise in transplant recipients—conditions that require activation of biopsychosocial resources to facilitate adaptation to the transplanted organ [6]. Health-related quality of life has increasingly become a key issue in terms of measuring outcomes after kidney transplantation, as any deterioration in post-transplant quality of life is associated with a high perception of infection risk and limited access to care [7]. Psychological well-being in kidney transplant recipients is influenced by other factors, including social support, resilience, and early attachment experiences within the family [8]. Among the psychological determinants that may affect mental health and quality of life, the concept of parental bonding has received growing attention in psychological research. Parental bonding refers to the quality of the emotional bond and care received from parents, particularly the mother, during childhood and has been identified as a predictor of long-term psychological well-being [9]. The model of parental bonding proposed by Parker [9] distinguishes between two primary dimensions: maternal care and maternal overprotection. Maternal care refers to affection, availability, and emotional support, while maternal overprotection involves excessive control behaviors that limit autonomy and psychological development. While maternal care is generally associated with good mental health, high maternal overprotection is often linked to psychological issues such as anxiety and depression [10].

Studies in general populations have shown that the quality of early attachment experiences significantly influences emotional regulation and coping strategies in adulthood [11]. Specifically, individuals who report experiencing loving and affectionate maternal care tend to develop greater psychological resilience and are better equipped to face life’s challenges. Conversely, relationships characterized by high maternal overprotection may interfere with emotional regulation, increasing vulnerability to psychological disorders. For example, research suggests that individuals with a history of overprotective parental care are at greater risk of developing mood disorders and anxiety in adulthood [12].

Despite these theoretical insights, few studies have investigated the impact of parental bonding on the quality of life and psychological health in kidney transplant recipients. Most research on kidney transplantation has focused on physical, medical, and social factors, while the importance of psychological factors related to early attachment experiences has been relatively overlooked. In the context of kidney transplant recipients, the quality of early attachment experiences, and particularly the bond with the mother, may play an important role in psychological adjustment to transplantation [13]. Findings from Calia [14] reported that confidence in relationships (a secure attachment dimension) was associated with better treatment compliance, while avoidant attachment was linked to poorer compliance. The authors suggested that assessing attachment style may help tailor psychological support to improve adherence in kidney transplant recipients.

However, exploring these factors could provide valuable insights into the development of psychosocial interventions aimed at improving patients’ psychological adjustment and, consequently, their overall quality of life [3]. Some studies suggest that psychological interventions, including the strengthening of emotional regulation and coping strategies, could improve transplant recipients’ psychological outcomes and, in turn, enhance their physical [3].

Hamama-Raz [8] found that attachment anxiety moderated the relationship between coping flexibility and illness acceptance: high coping flexibility was associated with greater illness acceptance only among those with low attachment anxiety.

Coskun [15] explored the relationship between transplant patients’ attachment styles and their QoL and psychological well-being post-transplantation. The results indicated that specific attachment styles (preoccupied and fearful) are correlated with the presence of depression, anxiety, and QoL of life features in liver transplant recipients. Understanding the transplant candidates’ attachment styles may be useful in identifying patients who might be at risk of experiencing depression or anxiety after liver transplantation.

These studies reported that insecure attachment may be associated with increased vulnerability to psychological distress and hindered adaptation post-transplant.

Specifically, no study has considered evaluating the parental bond in kidney transplant recipients and correlating this important psychological construct with the quality of life perceived by these subjects.

Therefore, our study aimed to explore how dimensions of parental bonding, particularly maternal care and overprotection, may influence the quality of life and psychological well-being in kidney transplant recipients. By investigating these relationships, the study seeks to understand whether early maternal attachment experiences can predict psychological outcomes in adult transplant recipients. This approach may offer new perspectives on improving psychosocial support for transplant patients, enhancing psychological resilience, and ultimately improving their quality of life after transplantation.

## 2. Methods

### 2.1. Participants and Procedure

The study involved 99 kidney transplant recipients who were recruited at the outpatient clinic of an Italian transplant center between May 2022 and July 2024 by telephone contact. All kidney transplant recipients undergoing follow-up at the Italian transplant center’s Clinical Psychology and Psychopathology Service were contacted by telephone. Of the 170 patients in the database, 30 refused to participate for personal reasons, 15 were deceased, and 26 never answered the phone calls. The initial phone call was just to explain the study and ask for participation. For those who joined the study, 1-2 interviews were performed in person to administer the questionnaires of the established protocol, using the interviewer-assisted administration method to maximize the response rate, ensure a lower number of missing answers, and reduce the difficulty of understanding the items. A psychiatric and psychological team—comprising a psychiatrist, a psychologist, and two trainee psychologists—assessed the participants during their first visit to the transplant clinic.

Inclusion criteria required participants to be over 18 years of age and to have undergone kidney transplantation at least 6 months prior. Exclusion criteria consisted of limited education (<5 years of schooling), psychiatric conditions such as Alzheimer’s disease, intellectual disability, or other cognitive impairments, and use of psychotropic medications (antipsychotics and/or antidepressants), as these factors could affect comprehension of assessment items.

Before enrollment, all participants received detailed information about the study’s aims and procedures and provided written informed consent. Participation was entirely voluntary, with no financial compensation offered.

The study was approved by the Local Ethics Committee (Comitato Etico “Catania 1”; approval code: 48908; approval date: 22 December 2020) and conducted in compliance with ethical standards of the Italian Psychological Association, the 1964 Declaration of Helsinki, and its subsequent amendments.

### 2.2. Measures

The tests administered during the interview were the following: Parental Bonding Instrument (PBI) to identify the type of parental bond; Short Form Health Survey-36 (SF-36) to evaluate the quality of life perceived by patients.

#### 2.2.1. PBI

Created in 1979 by researchers Parker, Tupling, and Brown, the PBI is one of the most widely used tools in psychology to assess individuals’ perception of the bond with their parents during childhood and adolescence. This self-administered questionnaire focuses on two fundamental dimensions of parenting: care, which reflects the degree of affection, emotional support, and availability shown by parents; and overprotection, which instead assesses the tendency to excessive control and to limit the child’s autonomy. The questionnaire is composed of 25 items (12 for the care scale and 13 for the overprotection scale), each rated on a 4-point Likert scale (from 0 = “not at all true” to 4 = “completely true”). A particularly interesting aspect of the PBI is its ability to classify parenting style into four distinct categories, based on the combination of scores obtained in the two dimensions: affectionate bond: parents who combine high levels of care with marked overprotection; optimal parents: high levels of care accompanied by low overprotection; affectionate control: low levels of care combined with high overprotection; and neglectful parents: low levels of both care and overprotection. To distinguish between “high” and “low” scores, the PBI uses specific cutoffs: for mothers, ≥27 points for care and ≥13.5 for overprotection; for fathers, ≥24 points for care and ≥12.5 for overprotection.

Numerous studies, including the original works by Parker [9,16,17], have demonstrated that the PBI has excellent psychometric properties, showing good reliability and validity.

#### 2.2.2. SF-36

The SF-36 is a 36-item questionnaire that assesses health status and perceived quality of life. It consists of 8 scales, obtained from the weighted sum of the responses in each section: Vitality (VT), Physical Functioning (PF), Bodily Pain (BP), Perception of General Health (GH), Physical Limitations (PR), Emotional Limitations (ER), Social Functioning (SR), and Mental Health (MH).

In addition, it provides two global indices: PCS (Physical Component Scale): physical health, and MCS (Mental Component Scale): mental health.

Scores range from 0 to 100 (higher values indicate better quality of life), with 50 as the reference value for all dimensions. Its reliability has been confirmed in patients with chronic kidney disease and transplant recipients [18,19,20].

### 2.3. Statistical Analysis

The statistical analysis was conducted using IBM SPSS Statistics version 29 [21]. The data obtained from this study were checked, and descriptive and inferential statistical analyses were then conducted. Pearson product–moment correlation coefficients were calculated to examine the relationships between the PBI and SF-36. Two hierarchical regression analyses were performed to investigate the association between health-related quality of life and parental bonds.

A theory-driven hierarchical approach was adopted. Each regression consisted of two steps: in Step 1, potential confounding variables (gender and duration of dialysis before transplantation) were entered; in Step 2, the four dimensions of parental bonding (maternal and paternal care and maternal and paternal overprotection) were entered simultaneously. The first regression used the SF-36 Physical component as the dependent variable, while the second regression used the SF-36 Mental component as the dependent variable. Assumptions for hierarchical regression analyses with physical and mental health as dependent variables were tested. Specifically, no violations were detected in terms of multicollinearity (Tolerance values: 0.565–0.944; VIFs: 1.060–1.771), normality of residuals (standardized residuals ranged from −2.345 to 2.515 for physical health and from −2.870 to 1.634 for mental health; both M = 0; SD = 0.968), linearity, homoscedasticity (both assessed visually through residual scatterplots), or independence of errors (Durbin–Watson = 2.108 for physical health; Durbin–Watson = 2.118 for mental health).

Gender was coded as a “dummy variable” in both regression analyses (“Male” = 1; “Female” = 2).

## 3. Results

### 3.1. Demographic Characteristics of the Sample

Table 1 shows the demographic characteristics of the final sample. The sample consisted of 99 kidney transplant recipients with a mean age of 52 years (*SD* = 9.93), ranging from 26 to 74 years. Regarding gender, 69.7% of participants were male (*n* = 69), while about half of the sample had completed primary or secondary school (*n* = 47, 47.5%). The most frequent medical causes of kidney failure were glomerulonephritis (*n* = 29, 29.3%) and polycystic kidney (*n* = 28, 28.3%). The mean time since diagnosis was 22.86 years (*SD* = 9.17), while the mean duration of dialysis before transplantation was 37.14 months (*SD* = 32.46). Additionally, the mean time since the transplant procedure was 7.72 years (*SD* = 5.17).

### 3.2. Descriptive and Correlational Analyses

Table 2 shows descriptive statistics and correlation coefficients among the study variables. The strongest correlation, a moderate negative association, was found between maternal overprotection and the SF-36 Physical component (*r* = −0.49, *p* < 0.01), indicating that higher levels of perceived maternal overprotection were associated with poorer physical health-related quality of life. The SF-36 Physical component was also moderately positively correlated with paternal care (*r* = 0.46, *p* < 0.05), suggesting that individuals perceiving higher paternal care reported better physical health-related quality of life. Additionally, maternal overprotection showed a weak positive correlation with paternal overprotection (*r* = 0.31, *p* < 0.01). Finally, mental health-related quality of life was positively correlated with maternal care (*r* = 0.28, *p* < 0.01) and negatively correlated with maternal overprotection (*r* = −0.28, *p* < 0.01).

### 3.3. Regression Analyses

Table 3 shows the results of the regression analysis for physical health in kidney transplant recipients. In Step 1, no variables were significant predictors. In Step 2, maternal care emerged as a significant positive predictor (*β* = 0.290; *p* < 0.05), indicating that higher levels of perceived maternal care during childhood are associated with better psychological health in these patients. Table 4 shows the results of the regression analysis for psychological health in kidney transplant recipients. In Step 1, no variables reached statistical significance. In Step 2, maternal overprotection was identified as a significant negative predictor (*β* = −0.286; *p* < 0.05), suggesting that higher levels of perceived maternal overprotection during childhood are associated with poorer psychological health in this patient population.

## 4. Discussion

### 4.1. Maternal Care and Quality of Life in Kidney Transplantation

The results of our study suggest that high maternal care is positively associated with better psychological health and quality of life in kidney transplant recipients, while high maternal overprotection is correlated with poorer psychological health and quality of life.

This focus on early attachment experiences in the context of kidney transplantation represents a novel contribution to the literature, as most prior research has concentrated on medical and social factors, with limited attention to the psychological roots of adaptation in this population.

These findings are consistent with studies showing that a mother–child relationship based on care and support can protect against the onset of psychological disorders and improve coping with chronic illness [22,23]. Studies focused on patients with chronic illnesses such as diabetes or cardiovascular diseases have shown that low-quality parental bonding, especially characterized by high overprotection, is associated with poorer psychological quality of life [24,25]. However, the literature on kidney transplant recipients remains relatively underdeveloped in this regard. While some studies have highlighted the importance of social support and psychological resilience in this group, the direct impact of parental bonding experiences has been less explored [3,26]. Our study addresses this gap by providing one of the first comprehensive examinations of how early attachment dynamics, particularly maternal care and overprotection, shape psychological outcomes in kidney transplant recipients.

### 4.2. The Role of Secure Attachment for Coping in Kidney Transplantation

Kidney transplant recipients continuously face uncertainty regarding their medical condition and must manage a complex disease process. Kidney transplantation requires not only physical recovery but also emotional adaptation to a new, uncertain medical status [27]. The ability to cope with ongoing medical care, immunosuppressive treatments, and potential complications may be shaped by early emotional experiences. A secure and supportive maternal relationship could enhance resilience and stress coping mechanisms, while an overprotective relationship could hinder the development of coping strategies, making it more difficult for the individual to manage the stress of living with a chronic illness [11,28,29]. The ability to cope with such stress is strongly influenced by psychological and social factors, including early attachment experiences [30].

Our findings underscore the unique psychological challenges faced by transplant recipients and highlight the need to consider early attachment experiences as a key factor in their long-term adaptation.

### 4.3. The Contribution of Our Study

Our study suggests that maternal attachment dynamics significantly influence psychological well-being post-transplant. In both regression models, the explained variance was relatively low. While statistically significant, these values suggest that other factors beyond maternal bonding may also influence the quality of life and psychological well-being of kidney transplant recipients. Future research should investigate these additional variables to gain a more comprehensive understanding of their impact.

Nevertheless, our study contributes important insights by suggesting that maternal attachment dynamics may have a significant impact on psychological well-being post-transplant. Our study offers inspiration for thought both to identify factors that may predict or moderate long-term psychological adaptation of kidney transplant recipients and to plan individualized psychotherapy pathways [6,31,32]. The psychological support for kidney transplant recipients could benefit from integrating interventions that consider the attachment history and family dynamics of patients. Specifically, healthcare professionals could assess the quality of family relationships, aiming to identify any patterns of excessive protection that might hinder the patient’s emotional autonomy. Family therapy or caregiver support programs might be helpful in improving the quality of family interactions and fostering a more balanced environment that promotes emotional independence [33,34,35]. Moreover, improving coping skills and psychological resilience through psychosocial interventions could help transplant recipients better manage the stress and challenges associated with post-transplant care [36,37]. Interventions should focus on strengthening the patient’s internal resources and enhancing their ability to cope with stressful situations, not only from a medical standpoint but also from a psychological perspective [38,39,40].

### 4.4. Clinical Implications

Patients who experienced adequate maternal care—characterized by secure attachment—tend to manage stress, resolve conflicts, and adapt to changes more effectively. While they may still experience transplant-related anxiety, they generally have higher self-esteem and emotional resilience. For these individuals, interventions could focus on enhancing coping skills, such as managing anxiety or fear of rejection, through therapies like cognitive-behavioral therapy (CBT). Even with a strong emotional foundation, psychological support can strengthen family relationships and monitor the quality of parent–child bonds. Family or couples therapy may also help maintain a robust support network [13,33].

In contrast, patients who experienced maternal overprotection during childhood may develop insecure attachment styles, leading to low self-esteem, difficulty managing autonomy, and heightened vulnerability to anxiety and stress. Overprotection can also result in excessive dependence on external support or difficulty separating from parents. Psychological interventions for these patients should focus on fostering emotional autonomy and decision-making skills. CBT can help reduce emotional dependency and address irrational fears exacerbated by overprotection. Family therapy may be particularly useful in addressing overprotective dynamics and improving family relationships. Interventions should also aim to build self-efficacy by reinforcing small successes and encouraging independence in daily choices [13,33].

Targeted psychological interventions for transplant recipients should consider individual differences in maternal care experiences and attachment styles. Patients with secure attachment and adequate maternal care may benefit from resilience-building interventions, while those with insecure attachment and overprotection may require support focused on autonomy, trauma management, and family dynamics. A personalized approach is essential to optimize post-transplant psychological outcomes [41,42]. This emphasis on tailored interventions based on attachment history represents an innovative approach to post-transplant care, moving beyond one-size-fits-all models.

### 4.5. Limitations

As a cross-sectional study, one of the primary limitations of this research is the inability to establish definitive causal relationships between parental bonding dimensions and psychological health. Although correlations between maternal attachment and psychological quality of life emerge, it is not possible to determine whether the quality of maternal attachment is a direct cause of psychological health outcomes or whether other unexamined variables may influence both dimensions. Additionally, the sample of 99 patients may not fully represent all the psychological and social factors influencing the quality of life in kidney transplant recipients. Longitudinal studies could provide deeper insights into the nature of these relationships over time, offering a clearer understanding of the causal mechanisms at play.

Furthermore, participants’ self-reports of their maternal attachment experiences could be influenced by memory biases or subjective interpretations of past experiences. Future research could benefit from the use of more objective measures or reports from parents to obtain a more balanced view of family dynamics.

## 5. Conclusions

Our study highlights the importance of maternal bonding for the psychological health and quality of life of kidney transplant recipients. The results suggest that maternal affection and support may serve as a protective factor, while excessive maternal protection could impair the development of emotional coping mechanisms necessary for dealing with the stresses of adult life. Psychosocial interventions aimed at improving psychological resilience and the quality of family relationships could represent an important opportunity to enhance psychological outcomes for transplant recipients. However, further longitudinal studies are needed to explore the causal dynamics between attachment and psychological quality of life in this population.

## Figures and Tables

**Table 1:** Demographic characteristics of patients included in the study (*n*= 99).

Variable	*M*	*SD*	*n*	%
**Age (in years)**	52	9.93		
**Gender**				
Male			69	69.7
Female			30	30.3
**Educational background**				
Primary or middle school diploma			47	47.5
High school diploma			43	43.4
Graduate			9	9.1
**Time since diagnosis (in years)**	22.86	9.17		
**Duration of dialysis before transplantation (in months)**	37.14	32.46		
**Time since the transplant procedure (in years)**	7.72	5.17		
**Original Nephrological disease**				
Polycystic kidney			28	28.3
Glomerulonephritis			29	29.3
Patient unable to recall the cause of kidney failure			22	22.2
Diabetes			8	8.1
Autoimmune disease (LES)			7	7.1
Congenital absence of one kidney			5	5.1

**Table 2:** Descriptive and correlational analyses (*n*= 99).

Variable	*Min*	*Max*	*M*	*SD*	*Skew*	*Kurt*	1.	2.	3.	4.	5.
1. PBI Maternal care	0	36	27.20	8.53	−1.22	1.20					
2. PBI Maternal overprotection	1	36	14.81	7.33	0.65	0.51	−0.49 *				
3. PBI Paternal care	6	36	24.49	8.14	−0.46	−0.62	0.46 *	−0.13			
4. PBI Paternal overprotection	0	36	12.66	7.07	0.81	0.66	−0.07	0.31 *	−0.07		
5. SF-36 Physical component	18	64	43.70	10.78	−0.43	−0.51	0.26	−0.19	0.10	−0.14	
6. SF-36 Mental component	19	62	45.35	10.80	−0.64	−0.65	0.28*	−0.28 *	0.09	0.02	−0.03

Note. PBI Parental Bonding Instrument, SF-36 Short Form (36) Health Survey, *Min* minimum value, *Max* maximum value, *M* mean, *SD* standard deviation, *Skew* skewness, *Kurt* kurtosis. For participants n. 47 and n. 90, the PBI Father data is missing because they did not know their father. For participant n. 75, the PBI Mother data is missing because he did not know his mother. * *p* < 0.01

**Table 3:** Standardized regression coefficients predicting physical health from maternal and paternal care and overprotection, controlling for gender and duration of dialysis before transplantation.

	Step 1	Step 2	*R* ^2^
Demographics			0.014
Gender	−0.060	0.020	
Duration of dialysis before transplantation	−0.103	−0.049	
Parental Bonding Instrument			0.114 *
Maternal care		0.290 *	
Maternal overprotection		−0.046	
Paternal care		−0.073	
Paternal overprotection		−0.130	

* *p* < 0.05.

**Table 4:** Standardized regression coefficients predicting mental health from maternal and paternal care and overprotection, controlling for gender and duration of dialysis before transplantation.

	Step 1	Step 2	*R* ^2^
Demographics			0.026
Gender	−0.154	−0.134	
Duration of dialysis before transplantation	0.045	0.126	
Parental Bonding Instrument			0.151 *
Maternal care		0.118	
Maternal overprotection		−0.286 *	
Paternal care		0.038	
Paternal overprotection		0.157	

* *p* < 0.05.

## Data Availability

The data that support the findings of this study are not openly available due to reasons of sensitivity and are available from the corresponding author upon reasonable request.
